# *Bapx1* upregulation is associated with ectopic mandibular cartilage development in amphibians

**DOI:** 10.1186/s40851-018-0101-3

**Published:** 2018-06-13

**Authors:** Paul Lukas, Lennart Olsson

**Affiliations:** Institut für Zoologie und Evolutionsforschung mit Phyletischem Museum, Ernst-Haeckel-Haus und Biologiedidaktik, Erbertstr. 1, 07743 Jena, Germany

**Keywords:** *Xenopus laevis*, *Ambystoma mexicanum*, Evolutionary novelties, Ly-294,002, *Nkx 3.2*, *Xbap*

## Abstract

**Background:**

The emergence of novel structures during evolution is crucial for creating variation among organisms, but the underlying processes which lead to the emergence of evolutionary novelties are poorly understood. The gnathostome jaw joint is such a novelty, and the incorporation of *bapx1* expression into the intermediate first pharyngeal arch may have played a major role in the evolution of this joint. Knockdown experiments revealed that loss of *bapx1* function leads to the loss of the jaw joint, because Meckel’s cartilage and the palatoquadrate fuse during development. We used *Xenopus laevis* and *Ambystoma mexicanum* to further investigate the function of *bapx1* in amphibians. *Bapx1* expression levels were upregulated through the use of Ly-294,002 and we investigated the potential consequences of the enhanced *bapx1* expression in amphibians to test the hypothesized joint inducing function of *bapx1*.

**Results:**

We show that Ly-294,002 upregulates *bapx1* expression in vivo. Additionally, ectopic mandibular arch derived cartilages develop after Ly-294,002 treatment. These ectopic cartilages are dorsoventrally oriented rods situated lateral to the palatoquadrate. The development of these additional cartilages did not change the muscular arrangement of mandibular arch-derived muscles.

**Conclusions:**

Development of additional mandibular cartilages is not unusual in larval anurans. Therefore, changes in the *bapx1* expression during evolution may have been the reason for the development of several additional cartilages in the larval anuran jaw. Furthermore, our observations imply a joint-promoting function of *bapx1*, which further substantiates its hypothetical role in the evolution of the gnathostome jaw joint.

## Background

The acquisition and incorporation of novel skeletal structures into an existing skeletal environment is a process that can cause morphological diversification. How such evolutionary novelties arose during evolution and under which circumstances they arose is an important question in evo-devo research. An important example is the evolution of the gnathostome jaw and its skeletal diversification in different phyla. The jaw itself consists basically of a dorsal and a ventral element, which are connected by a distinct joint [1]. Jaw evolution enables predation on large and motile prey and can therefore be seen as one of the central innovations in gnathostomes.

The larval anuran jaw is distinct from those in other vertebrates. At the base of anurans two additional jaw structures, the rostralia, evolved. The suprarostral cartilage forms the anterior part of the upper jaw, whereas the infrarostral cartilage is part of the lower jaw and movable against Meckel’s cartilage via the intramandibular joint [2]. These two additional structures made possible the evolution of numerous different feeding modes in anuran tadpoles, which enabled the decoupling of larval and adult stages. The diverse feeding modes, which are based on the derived morphology, may be a contributing factor behind why anurans are the dominant recent amphibian group [3]. During further evolution several anuran taxa evolved one or more additional cartilages within the larval jaw. Such adrostral cartilages are described in *Heleophryne natalensis*, *Pelobates fuscus*, *Alytes obstetricans* and *Pelodytes punctatus*, to just name a few species [4, 5]. Three different mechanisms for the evolution of such additional cartilaginous structures have been proposed by Svensson and Haas [3]: (1) through duplication of existing cartilages; (2) through partitioning of existing cartilages through the development of new joints; (3) through de novo evolution of cartilages not homologous to existing elements. In all three cases, one or more genetic regulators must be responsible for the evolution of the novel cartilage.

*Bapx1* (also known as *nkx3.2*and *xbap*) homologues are present in different, distantly related phyla. This gene was first identified in *Drosophila*, where it spatially subdivides the mesoderm and is thus essential for midgut musculature formation [6]. In amphioxus and lamprey *bapx1* is expressed in the pharyngeal endoderm [1, 7]. During pre-gnathostome evolution *bapx1* is suggested to have been incorporated into an existing pharyngeal arch patterning system [1]. In such a system, a gradual expression of homeobox genes defines an anterior-posterior axis. Overlapping expression of these genes defines different regions along this axis [8–10]. For instance, the first pharyngeal arch is defined by the absence of *hox* expression, whereas the second pharyngeal arch is defined by *hoxa2* expression [11, 12]. Gnathostome pharyngeal arches are patterned dorsoventrally by a nested expression of *dlx* genes [13, 14]. These two patterning programs together form a developmental grid that enables locally restricted gene expression dependent on the specific spatial configuration. The incorporation of *bapx1* into this pre-gnathostome head patterning program has been suggested to have played a major role in the evolution of the gnathostome jaw [1, 15]. In lamprey, which lacks a dorsoventral patterning mediated by *dlx* genes, *bapx1* is not expressed in the first pharyngeal arch, whereas in *Scyliorhinus* and zebrafish *bapx1* expression dependent on *dlx* function within the first pharyngeal arch has been reported [16–18]. In zebrafish *bapx1* expression can be found in an intermediate domain of the first pharyngeal arch and is ventrally restricted by a *barx1* expressing domain [17, 19]. *Barx1* expression is ventrally restricted by *hand2* expression which inhibits *bapx1* expression during development [19]. In zebrafish *bapx1* is expressed in the intermediate domain of the first pharyngeal arch, exactly where the primary jaw joint will form. Homologous genes with similar first pharyngeal arch expression can be found in *Xenopus* [20], *Pleurodeles* [21], chicken [22], mouse [23] and human [24]. *Bapx1* knockdown in zebrafish led to fusion of Meckel’s cartilage and the palatoquadrate, resulting in loss of the primary jaw joint [17]. The same result is seen after downregulation of *bapx1* in amphibians (Lukas and Olsson, submitted) indicating a role for *bapx1* in both development and evolution of the primary jaw joint. Loss of *barx1* function in zebrafish led to dorsal expansion of *hand2* expression and the formation of an ectopic joint within Meckel’s cartilage where *hand2* and *bapx1* expression domains met [19]. This ectopic joint development after *barx1* inactivation and the following expansion of the *bapx1* expression domain further indicates that *bapx1* expression can induce joint development in the first pharyngeal jaw.

The role of phosphatidylinositol 3-kinase (PI3K) in cell metabolism, regulation of gene expression, cell survival, and cell growth is well-documented [25, 26]. It has been shown that PI3K signaling can down-regulate *bapx1* specifically by using the catalytic subunit p85β in mice [27]. *Pik3ca*, the PI3K subunit responsible for the *bapx1* suppression pathway, is expressed in *X. laevis* in the pharyngeal region from NF 26 to NF 32 [28]. Ly-294,002 (2-(4-Morpholinyl)-8-phenyl-4H-1-benzopyran-4-one hydrochloride) is a specific inhibitor of PI3K [29]. PI3K suppression mediated by Ly-294,002 causes elevated *bapx1* expression levels [27]. To test the effects of overexpression of *bapx1* in the development of the first pharyngeal arch in amphibians, Ly-294,002 was used in this study to inhibit *pik3ca* function and enhance *bapx1* expression in vivo.

## Methods

### Amphibian husbandry

Males and females of *Xenopus laevis* (Daudin) and *Ambystoma mexicanum* (Shaw) were kept in separate groups in our breeding colony in Jena. Adults and larvae of *Xenopus laevis* were kept at 22 °C. To induce mating and obtain fertilized eggs *Xenopus laevis* adults were put pairwise into darkened basins with lowered water level. They were kept there over night at 16 °C. After successful egg deposition the eggs were collected and then dejellied using a solution of 2% cysteine hydrochloride. The eggs were washed several times and cultured in 0,1× modified Barth’s saline (MBS) with 50 μg/ml gentamycin at 22 °C. *Ambystoma mexicanum* adults were kept at 18 °C. Single pairs were transferred into basins with fresh water and ice was added to lower the temperature and induce mating overnight. After successful egg deposition and fertilisation the eggs were collected and manually dejellied using dissecting forceps. The eggs were cultured in 20% Steinberg’s solution with 50 μg/ml gentamycin at 22 °C. Developmental stages were determined according to Nieuwkoop and Faber [30], Ziermann and Olsson [31] for *X. laevis* and Schreckenberg and Jacobson [32] for *A. mexicanum*. Nieuwkoop and Faber (NF) staging was used for the identification of early developmental stages. For the description and comparison of the inner morphology of treated embryos Ziermann and Olsson staging (ZO) was used because this staging table provides more comparable stages during chondrification and initial skeletal development than does Nieuwkoop and Faber.

### In vivo experiments

Embryos of *X. laevis* were incubated with different concentrations of LY-294,002 hydrochloride (Merck) at different developmental stages. LY-294,002 was initially dissolved in dimethyl sulfoxide (DMSO). A stock solution containing 10 μM DMSO and 1 mM LY-294,002 was prepared and diluted with MBS to concentrations of 10 μM, 20 μM, 30 μM, 40 μM and 50 μM LY-294,002. *X. laevis* embryos (*N* = 90 for each stage and concentration) were then incubated at NF 10 (onset of gastrulation), at NF 13 (onset of neurulation), at NF 22 (early tailbud stage), at NF 29 (late tailbud stage) and NF 39 (onset of cartilage development) in the different LY-294,002 dilutions. *X. laevis* embryos were also kept in 1× MBS and 0,1 μM DMSO in 0,1xMBS as a control. To specifically test the influence of LY-294,002 on mandibular arch development, 10 nl of 20 μM LY-294,002 was injected in the area of the mandibular neural crest segment [33] antero-ventral to the eye at NF 29. Injection was performed in 4% Ficoll/ 0,1× MBS and after 4 h the embryos were transferred into 0,1× MBS. *A. mexicanum* embryos were incubated with 20 μM Lys-294,002 at Schreckenberg and Jacobson stage (SJ) 36. *X. laevis* embryos were cultured until they reached NF 45 and *A. mexicanum* embryos were cultured for 5 days. The 0,1 μM DMSO and the 0,1xMBS solution as well as the different Lys-294,002 solutions were changed daily. Living, dead and malformed larvae were counted and images were taken using a Zeiss Stemi SV11 and an attached camera (ColorView) operated by AnalySIS software. Anaesthesia was performed using 1% tricaine methanesulfonate (MS-222) according to the animal welfare protocols at the Friedrich-Schiller-Universität Jena. Depending on further investigations larvae were fixed in 4% phosphate-buffered formalin (PFA), Dent’s fixative or RNA stabilisation reagent.

### Tissue staining

PFA-fixed larvae were dehydrated and embedded in paraffin. Serial sectioning was performed using a rotary microtome (Microm, HM 355 S). Sections of 7 μm thickness were obtained and subsequently stained with Heidenhain’s Azan technique [34] or nuclear fast red staining [35]. Images were taken with an XC10 Olympus camera mounted on an Olympus BX51 microscope operated with dotSlide software. *X. laevis* and *A. mexicanum* larvae fixed with Dent’s fixative was used for whole mount antibody staining. Cartilage cells were specifically stained with a monoclonal antibody against collagen II (II6B3-collagen II). A polyclonal antibody against newt skeletal muscle (12/101) was used to specifically stain muscle cells. For the colour reaction secondary antibodies conjugated with Alexa 488 and Alexa 568 (Molecular Probes) were used. The specimens were scanned with a confocal laser scanning microscope (Zeiss LSM 510) operated with Zen software. The image stacks obtained were further processed with Amira 6.0.1 (surface render) and Autodesk Maya® 2017 (rendering).

### In situ hybridisation and quantitative PCR

Whole-mount in situ hybridisations were carried out according to the protocol in Harland (1991) with a few modifications described by Square et al. [36]. NF 30–40 larvae were treated for 15 min and NF 40–45 larvae were treated for 20 min with Proteinase K. BM-Purple (Roche) was used for signal development. Probe of *bapx1* was kindly provided by Jennifer Schmidt. RNA from whole embryos kept in 0,1× MBS, in 0,1 μM DMSO and different Ly-294,002 solutions was isolated using QIAzol Lysis Reagent (Qiagen) and purified using RNeasy Mini Kit (Qiagen) according to manufacturer’s instructions. RevertAid Transcriptase (Thermo Scientific) was used to synthesize complementary DNA from 2 μg RNA extracted from embryo. Quantitative PCR was performed using a Stratagene Mx3005P (Agilent Technologies) and one- step qPCR SYBR green kit (Roche). Sequences of primers used for amplification of *bapx1* were taken from Square et al. [36]. Target gene expression was normalized [37] to the expression level of histone H4 (5’-GACGCTGTCAACCGAG-3’and 5’- CGCCGAAGCCAGAGTG-3’).

## Results

### Effect of Ly-294,002 treatment on tadpole survival

**Fig. 1:**
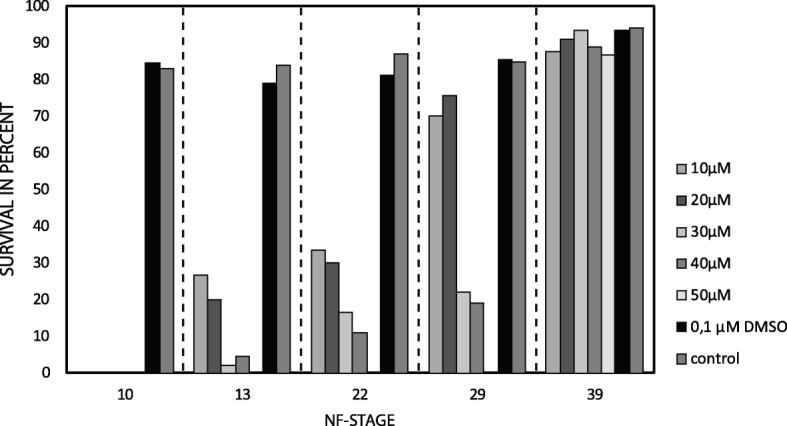
Quantitative analysis of *X. laevis* tadpole survival after Ly-294,002 treatment. *X. laevis* larvae were treated with different amounts of Ly-294,002 at different developmental stages until NF 45. As controls, *X. laevis* larvae were raised either in 0,1 μM DMSO or in 0,1× MBS. Larvae treated with DMSO and with 0,1× MBS show no significant differences in survival rates. The survival rate in Ly-294,002 treated larvae declines with increasing Ly-294,002 concentration and early start of the incubation

Initially embryos of *X. laevis* were treated with different concentrations of Ly-294,002 at different developmental stages. No embryos incubated at NF 10 survived the treatment with the different amounts of Lys-294,002 (Fig. 1). Embryos treated with 10 μM and 20 μM at the onset of neurulation (NF 13) survived the treatment at moderate rates (27 and 20% respectively) whereas only a minority of embryos treated with 30 μM (2%) and 40 μM (4%) survived. No embryo survived the treatment with 50 μM Ly-294,002. Survival rates slightly increased when embryos were reared in different Ly-294,002 solutions at NF 22. As before, no embryo survived treatment with 50 μM Ly-294,002. Larvae incubated at the late tailbud stage (NF 29) showed a much higher survival rate at lower Ly-294,002 concentrations. 70% of the larvae survived the treatment with 10 μM and 76% survived the treatment with 20 μM Ly-294,002, whereas no larvae survived the treatment with 50 μM. Larvae reared in the different concentrations of Ly-294,002 at the onset of chondrification (NF 39) show almost normal survival rates. No significant difference in the survival rate at the different developmental stages was observed between embryos reared in 0,1 μM DMSO and embryos reared normally in 0,1× MBS. Additionally, no abnormalities developed after DMSO treatment (Fig. 1). These circumstances indicate that changes in survival rate and morphology are not the result of the necessary presence DMSO in Ly-294,002 in vivo experiments and indicate a specific effect of Ly-294,002.

### Ly-294,002 treatment induces development of an ectopic mandibular cartilage

**Fig. 2:**
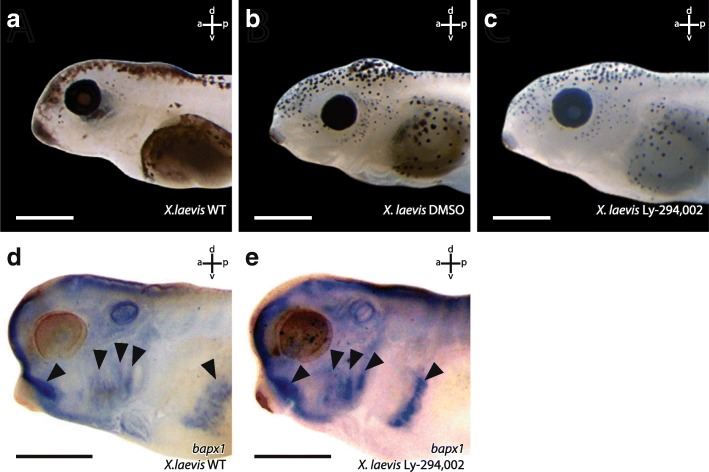
Effects of Ly-294,002 treatment on external head morphology and *bapx1* expression in *X. laevis*. Lateral view of larvae at NF 44 after rearing in **a** 0,1× MBS as control, **b** 0,1 μM DMSO and **c** 20 μM Ly-294,002 from NF 29 onwards. No significant differences in the external morphology can be observed. In situ hybridisation of NF 37 *X. laevis* larvae raised in **d** 0,1xMBS as control and **e** 20 μM Ly-294,002 from NF 29 onwards in lateral view reveal differences in the expression of *bapx1* in the mandibular arch. After *bapx1* upregulation the mandibular arch expression domain is broader and extends postero-dorsally. Scale bar 500 μm

**Fig. 3:**
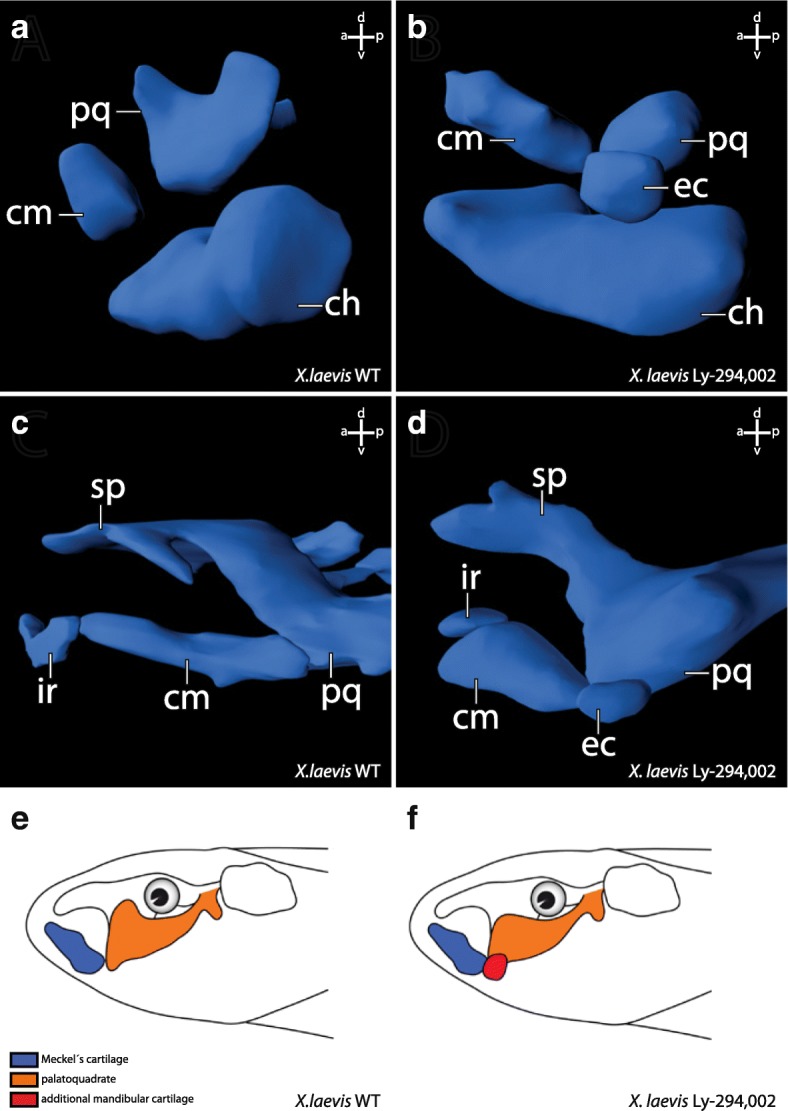
3D reconstructions based on confocal laser scanning microscopy showing the effects of Ly-294,002 treatment on cranial cartilage morphology in *Xenopus laevis* larvae. Lateral view of ZO 10 (**a**, **b**) and ZO 17 (**c**, **d**) larvae with normal *bapx1* expression (left column) and elevated *bapx1* expression (right column). In Ly-294,002-treated specimen with increased *bapx1* expression an ectopic cartilage develops within the mandibular arch lateral to the palatoquadrate. Depictions of *X. laevis* larvae in lateral view show the main differences between **e** normal and **f** perturbed development. In short, the processus muscularis is reduced in size and an ectopic cartilage arises lateral to the palatoquadrate after upregulation of *bapx1*. ch, ceratohyal; cm, Meckel’s cartilage; ec, ectopic cartilage; ir, infrarostral cartilage; pq, palatoquadrate; sp., suprarostral plate

**Fig. 4:**
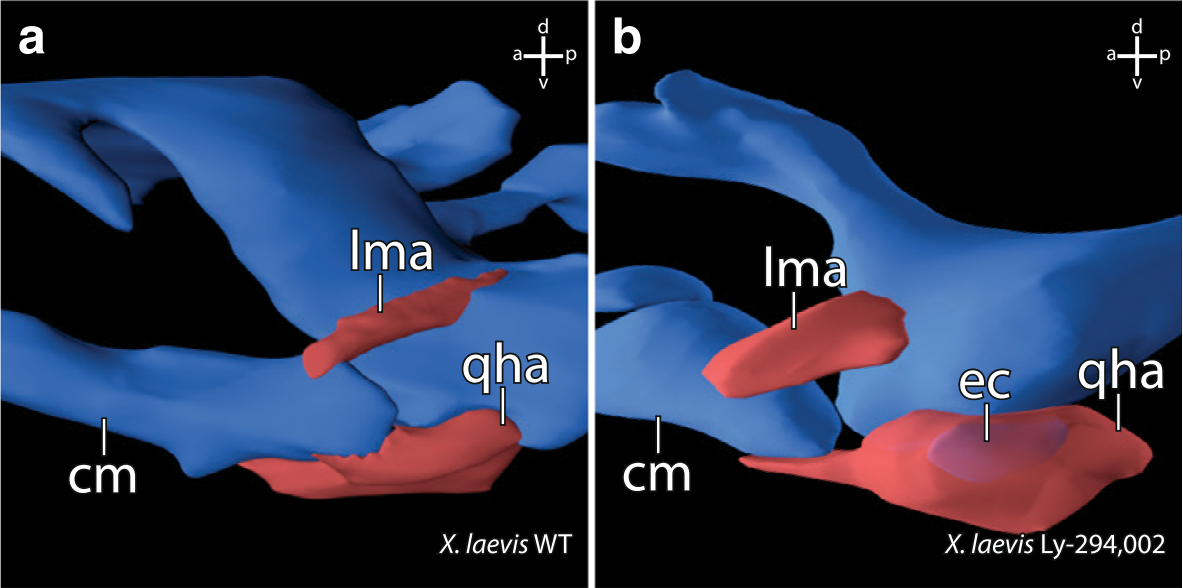
3D-reconstructions based on confocal laser scanning microscopy of specimen stained with antibodies showing the effects of *bapx1* upregulation on cranial muscle morphology in *Xenopus laevis* larvae. Lateral view of the jaw articulation and selected muscles of ZO 17 larvae. **a** Muscle morphology in a control specimen. **b** Muscle morphology in a specimen after *bapx1* upregulation. The m. levator mandibulae articularis originates more anteriorly on the dorsal surface of the processus muscularis than in control specimens. The m. quadratohyoangularis surrounds the ectopic cartilage without originating from or inserting onto this cartilage. cm, Meckel’s cartilage; ec, ectopic cartilage; lma, M. levator mandibulae articularis; qha, M. quadratohyoangularis

The external morphology of all surviving *X. laevis* larvae from these incubation experiments was checked for potential morphological changes. We did not observe any external morphological or behavioural abnormalities in either control larvae, larvae raised in 0,1 μM DMSO or in Ly-294,002 treated larvae (Fig. 2a-c). We also checked for internal musculoskeletal changes. No such changes were observed in larvae treated with the different concentrations of Ly-294,002 at NF 10, NF 13, NF 22 or NF 39. Interestingly, larvae treated at NF 29 that survived this procedure developed changes in the mandibular skeleton. The highest rate of larvae with disturbed mandibular arch morphology was seen after treatment with 20 μM Ly-294,002 at NF 29 (Table 1). Of the larvae which survived the treatment, 57% developed an additional mandibular cartilage, whereas in larvae treated with other concentrations of Ly-294,002, lower rates of this mandibular arch phenotype were observed (Table 1). For further analyses, we used 20 μM Ly-294,002 and treated larvae from NF 29 onwards. At ZO 10 (NF 40–41) the palatoquadrate, Meckel’s cartilage and the ceratohyal were present in unperturbed larvae (Fig. 3a). In treated larvae an ectopic cartilage with a rounded shape was visible lateral to the palatoquadrate and Meckel’s cartilage. This ectopic cartilage was bordered postero-dorsally by the palatoquadrate and antero-dorsally by Meckel’s cartilage. The ectopic cartilage was separated from the two cartilages by a cavity-like gap (Fig. 3b). During further development the rounded shape and the location of the ectopic cartilage remained the same (Fig. 3d). At ZO 17 (NF 44) Meckel’s cartilage is normally sigmoidally elongated (Fig. 3c) but in treated larvae Meckel’s cartilage was shortened and thicker than in controls (Fig. 3d). The suprarostral plate bent more dorsally than normal and the muscular process had partly lost its dorsal projection in treated larvae. The area where the ectopic cartilage arose during development was nearly the same in all treated larvae. The changed morphology seemed to have no large effect on the behaviour because treated larvae showed normal feeding and respiration. Next, we investigated whether mandibular muscles displayed any abnormalities and whether muscles inserted onto or originated from the ectopic cartilage. We observed only two muscles which are affected by the Ly-294,002 treatment. The M. levator mandibulae articularis normally originates from the posterior-ventral surface of the muscular process of the palatoquadrate and inserts onto the dorsal surface of the posterior end of Meckel’s cartilage (Fig. 4a). In perturbed larvae the muscle originated more anteriorly from the muscular process of the palatoquadrate and inserted more laterally and anteriorly onto Meckel’s cartilage than in controls (Fig. 4b). The M. quadratohyoangularis normally originates from the ventral surface of the palatoquadrate and from the dorsal surface of the ceratohyal and inserts onto the ventro-lateral edge of Meckel’s cartilage (Fig. 4a). In perturbed larvae origination and insertion were similar, but the insertion onto Meckel’s cartilage was smaller than in controls. The dorsal portion of this muscle surrounded the ectopic cartilage, but not even a single fibre inserted onto or originated from this cartilage (Fig. 4b).

**Table 1:** Frequency of mortality and phenotype occurrence in *Xenopus laevis* after treatment with different concentrations of Ly-294,002 from 29 NF onwards

	Concentration	Mortality	Phenotype
Control	–	15%	0%
DMSO	0,1 μM	14%	0%
Ly294 (29NF)	10 μM	30%	43%
	20 μM	24%	57%
	30 μM	78%	35%
	40 μM	81%	18%
	50 μM	100%	0%

Phenotypes were determined 6 days after incubation based on mandibular arch abnormalities including the appearance of ectopic cartilages. *n* = 90 for controls and each concentration of Ly-294,002

### Ly-294,002 treatment upregulates *bapx1* expression levels

**Fig. 5:**
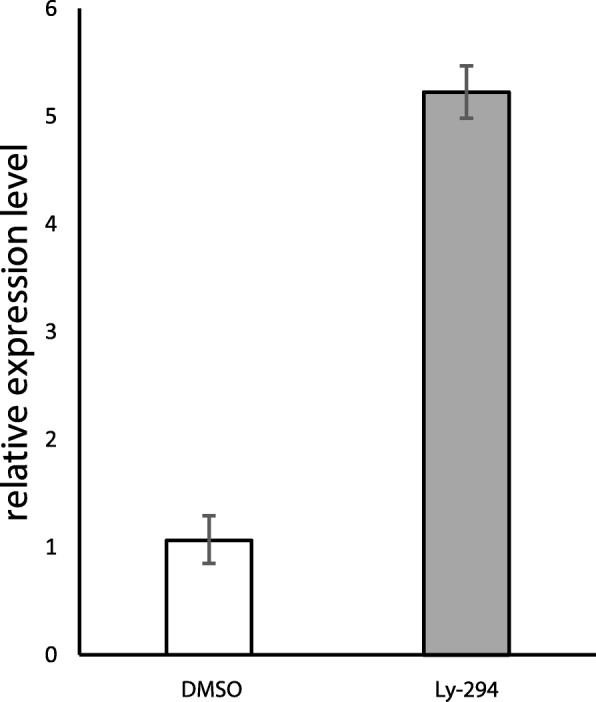
Effect of Ly-294,002 treatment on *bapx1* expression in *Xenopus laevis* at NF 45. Expression levels were determined through relative quantification (Livak method). Error bars indicate standard deviation of PCR runs (*n* = 3). DMSO reared specimens show no significant reduction in expression compared to control specimens. Ly-294,002 treated specimens show a ~ 5 times higher expression level of *bapx1* in comparison to controls

To test if the development of mandibular ectopic cartilages is correlated with changes in *bapx1* expression, we used quantitative PCR to investigate the relative expression of *bapx1*. We checked the expression levels for larvae reared in 0,1 μM DMSO, to investigate the effect of DMSO on *bapx1* expression, and for larvae reared in 20 μM Ly-294,002 from 29NF onwards. Rearing larvae in 0,1 μM DMSO had no effect on *bapx1* expression levels (Fig. 5 white bar). The unaltered *bapx1* expression in tadpoles raised in 0,1 μM DMSO further confirmed that DMSO did not interfere with our experiments. The inhibition of PI3K mediated by Ly-294,002 treatment raised the expression levels of *bapx1* more than fivefold (Fig. 5 dark grey bar). To further check for differences in the spatial expression we used whole mount in situ hybridisation of *bapx1* transcripts. Normally, *bapx1* was expressed in the ventral region of the mandibular arch at NF 37. The expression domain surrounded the cement gland dorsolaterally and marked the precursors of the palatoquadrate and the proximal part of Meckel’s cartilage (Fig. 2d). Posteriorly, *bapx1* expression was visible in the endoderm of the pharyngeal pouches of pharyngeal arches 3–5. More posteriorly *bapx1* was expressed in the foregut (Fig. 2d). In larvae treated with 20 μM Ly-294,002 from NF 29 onwards *bapx1* expression was visible in the same regions as described for the control larva (Fig. 2e). No duplicated expression domain was visible. The expression domain in the mandibular arch was broader than in the control larvae and extended more posteriorly. Treatment with Ly-294,002 led to increased *bapx1* expression levels and to an extension of the *bapx1* expression domain in the mandibular arch.

### Ectopic cartilage development after mandibular arch *bapx1* upregulation

**Fig. 6:**
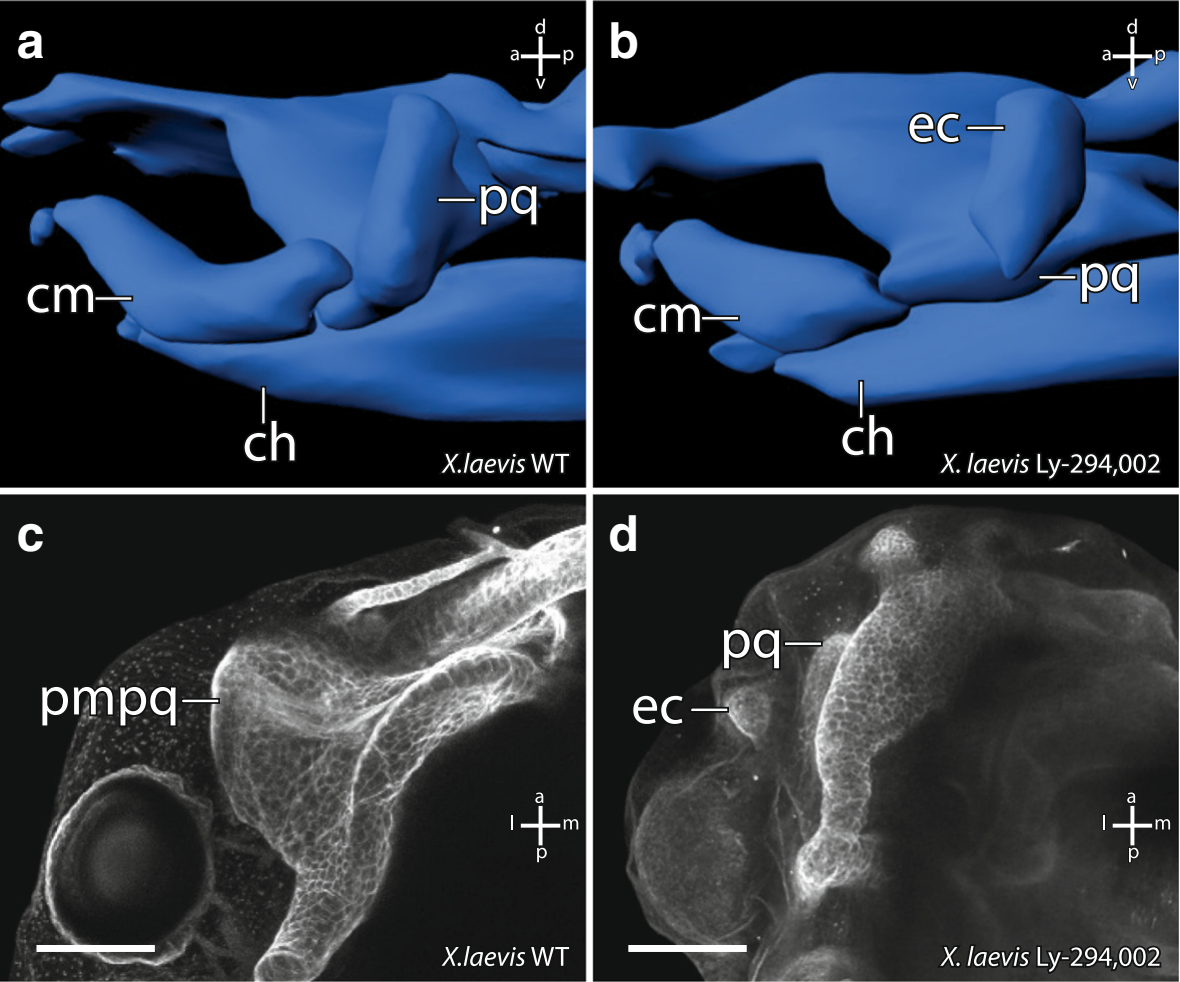
Effect of Ly-294,002 injection into mandibular arch precursors on cartilaginous cranial morphology in *X. laevis* larvae. 3D-reconstructions based on confocal laser scanning microscopy of **a** control and **b** injected specimens at ZO 17 in lateral view. Maximum intensity projections of **c** control and **d** injected specimens at ZO 20 stained with monoclonal II6B3-collagen II antibody and Alexa 568 in dorsal view. The reduction of the processus muscularis and the presence of an ectopic cartilage can be observed in injected specimens. ch, ceratohyal; cm, Meckel’s cartilage; ec, ectopic cartilage; pmpq, processus muscularis of the palatoquadrate; pq, palatoquadrate. Scale bar 200 μm

To investigate whether the observed cartilaginous changes in *X. laevis* are correlated to higher *bapx1* expression in the mandibular arch, we injected 20 μM Ly-294,002 into the area of the mandibular crest identified by Sadaghiani and Thiébau (1987) at NF 29. In the Ly-294,002-treated specimen an ectopic cartilage occurred more posterior than in larvae reared in Ly-294,002 solution. Posterior to the primary jaw joint articulation, a dorsoventral elongated cartilaginous rod was visible at ZO 17. It seemed to comprise cells from the muscular process of the palatoquadrate, because the process was reduced in size and lacks the dorsal projection seen in controls (comp. Fig. 6a and b). The ectopic cartilage was embedded in connective tissue and no muscle inserted onto or originated from its surface. Furthermore, the ectopic cartilage was distantly located in relation to Meckel’s cartilage and the palatoquadrate and did not articulate with any of these cartilages (Fig. 6d). During further development, the muscular process of the palatoquadrate slightly extended its surface dorsally and formed a miniaturized process compared to unperturbed larvae at ZO 20 (NF 45–46; comp. Fig. 6c and d). The ectopic cartilages remained as dorsoventrally elongated rod-like structures lateral to the muscular process of the palatoquadrate.

### Ly-294,002 treatment in axolotl

**Fig. 7:**
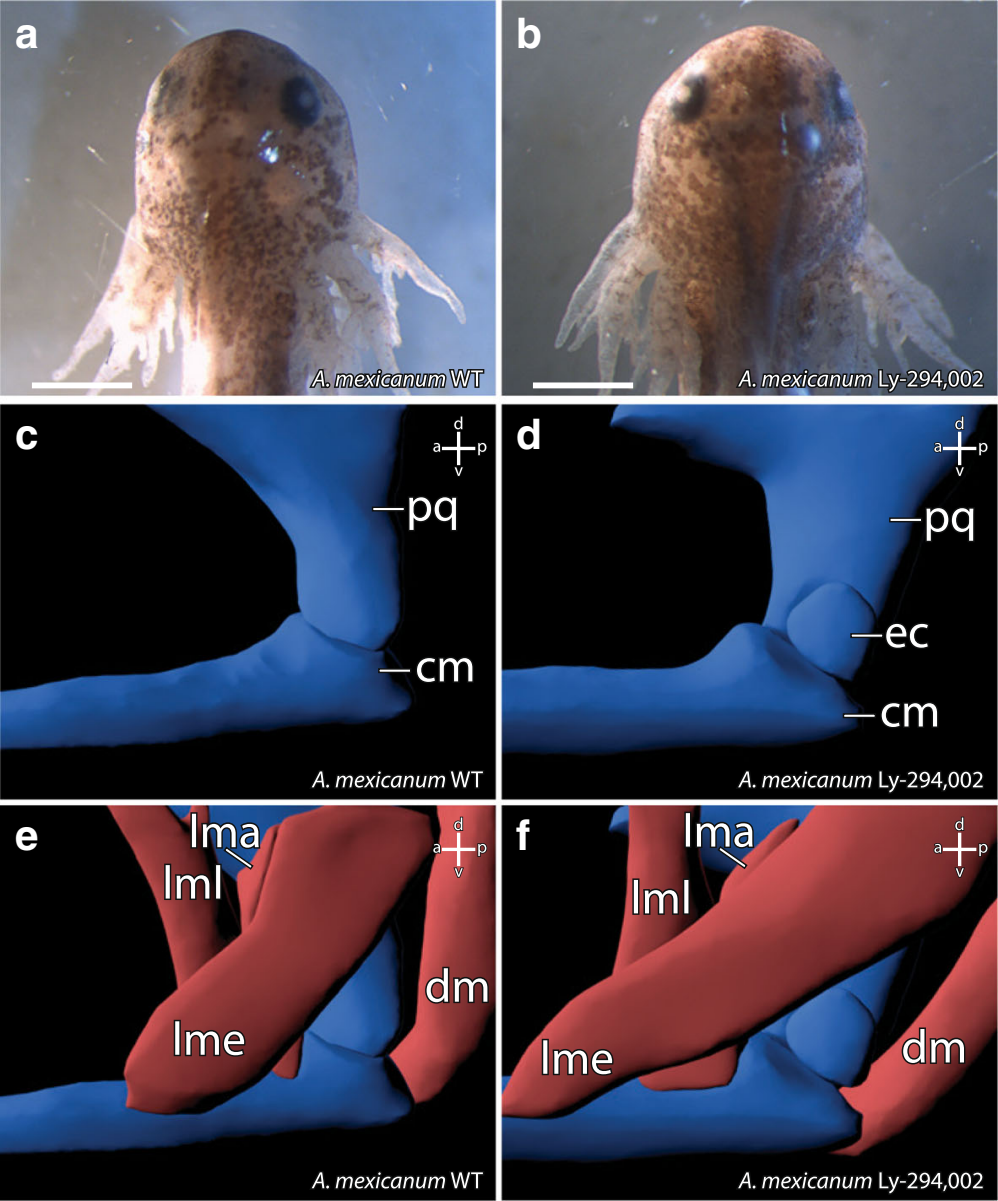
Effect of Ly-294,002 treatment on the external and internal morphology of *A. mexicanum*. Dorsal view of **a** control and **b** Ly-294,002 treated specimens reveals no differences in external morphology. Lateral view of 3D-reconstructions based on confocal laser scanning microscopy of **c** control and **d** Ly-294,002 treated specimen shows the development of an ectopic cartilage lateral to the jaw articulation in treated specimens. The musculature of **e** control and **f** treated specimens are similar and no changes in origination or insertion of muscles can be observed after *bapx1* upregulation. cm, Meckel’s cartilage; dm, M. depressor mandibulae; ec, ectopic cartilage; lma, M. levator mandibulae articularis; lme, M. levator mandibulae externus; lml, M. levator mandibulae longus; pq, palatoquadrate. Scale bar 500 μm

We have observed that Ly-294,002 treatment upregulates *bapx1* expression in *X. laevis*, and that the treatment simultaneously led to the development of a mandibular ectopic cartilage. Next, we sought to repeat the experiments with another amphibian to test whether the obtained results are a consequence of the derived state of *X. laevis* or represent a common feature among amphibians. Therefore, we investigated the effects of Ly-294,002 treatment on the development of *A. mexicanum*. We treated *A. mexicanum* larvae at SJ 36, which approximately corresponds to *X. laevis* NF 29, with 20 μM Ly-294,002 and reared them for 5 days. As in *X. laevis*, no significant changes in external morphology were observed (comp. Fig. 7a and b). However, the cartilaginous structures of the mandibular arch were malformed in ways similar to the case in *X. laevis*. Postero-dorsal to Meckel’s cartilage and latero-ventral to the palatoquadrate an ectopic cartilage occurred in treated larvae (Fig. 7d). This cartilage seems to be separated from Meckel’s cartilage and the palatoquadrate and was rounded in shape. Ventrally, the palatoquadrate was broader and narrower than in control larvae (comp. Fig. 7c and d). Laterally it had lost contact to Meckel’s cartilage and only articulated medially with it. Meckel’s cartilage also forfeited lateral contact to the palatoquadrate and only articulated medially. The lateral side of the posterior end seemed to articulate with the ectopic cartilage. The insertion and origination of the different muscles required for proper jaw movement were not affected by the Ly-294,002 mediated *bapx1* upregulation (comp. Fig. 7e and f). No such muscle inserted on the ectopic cartilage (Fig. 7f).

## Discussion

High doses of the PI3K-inhibitor Ly-294,002 and incubation at early stages (beginning of gastrulation, beginning and end of neurulation) lead to dramatically decreased survival rates in treated specimens (Fig. 1). These decreased survival rates may be a consequence of the major role of PI3K in various developmental processes such as cell growth, cell differentiation and cell survival [25, 26]. High doses of Ly-294,002 at early developmental stages may restrict the function of PI3K in cell growth and survival which leads to premature death. When incubated at relatively low concentrations of Ly-294,002 the PI3K regulated key processes probably proceed normally, whereas the inhibition of *bapx1* regulation remains disturbed. Between NF 29 and NF 39 the sensory organs develop, first muscle anlagen develop and differentiate into muscle fibres, the nervous system develops and excretory as well as digestive systems forms [30]. Ly-294,002 treatment during this period and earlier has a very large influence on mandibular arch development. We have shown that treatment with 20 μM Ly-294,002 from NF 29 onwards significantly elevates the expression of *bapx1* in *X. laevis* larvae using quantitative PCR. This upregulation additionally leads to a posterior expansion of the *bapx1* expression in the mandibular arch primordia as visualized through in-situ hybridisation. Furthermore, we have shown that DMSO, which was used to dissolve Ly-294,002, has no effect on tadpole survival and does not influence *bapx1* expression.

### Elevated *bapx1* expression is correlated to subdivision of existing cartilage

The treatment with Ly-294,002 in *X. laevis* and *A. mexicanum* has an effect on the development of mandibular arch derived cartilaginous elements. Neither in incubation nor in injection experiments were morphological changes of the non-mandibular arch derived cartilaginous skeletal elements observed. In both species tested, an ectopic cartilage develops after Ly-294,002 treatment. This cartilage appears lateral to the palatoquadrate and postero-lateral to Meckel’s cartilage (Fig. 6b). In *X. laevis* it is clearly visible that the lateral projection of the palatoquadrate, the muscular process, is reduced after Ly-294,002 treatment (Fig. 6d). The location of the ectopic cartilage suggests that the chondrocytes which form the ectopic cartilage became separated from the palatoquadrate during early development. These separated chondrocytes together may have formed a new lateral condensation which developed into the ectopic cartilage. Normally the m. orbitohyoideus and the m. quadratohyangularis originate from the lateral edge of the muscular process, but their origin is not the ectopic cartilage which is assumed to be formed by chondrocytes from the muscular process. Instead, they originate from the small remains of the muscular process. The chondrocytes which form the ectopic cartilage might have lost the ability to attract muscle progenitor cells. Additionally, no muscle inserts onto this ectopic cartilage, which indicates that no identity shift has taken place that would cause a muscle to shift its insertion. In *A. mexicanum* chondrocytes which normally form the lateral part of the palatoquadrate and the postero-dorsal part of Meckel’s cartilage become separated during development and seem to form the ectopic cartilage after *bapx1* upregulation. An indentation is visible where chondrocytes are missing at both Meckel’s cartilage and the palatoquadrate. Just as in *X. laevis*, no muscle originates from or inserts into the ectopic cartilage. In both species the ectopic cartilage appears to consist of chondrocytes that originate from existing cartilaginous structures. The mandibular arch derived cartilaginous structures, which are situated next to the ectopic cartilage, are reduced in size where they adjoin to the ectopic cartilage. We suggest that the ectopic cartilage cannot be characterized as a de novo developed cartilage, because Meckel’s cartilage and the palatoquadrate are reduced in size and developed altered shape after Ly-294,002 treatment, which indicates that the ectopic cartilage comprises of chondrocytes separated from these cartilages. The development of the ectopic cartilage as a consequence of the Ly-294,002 treatment is correlated to the raised *bapx1* expression levels which are also caused by the Ly-294,002 treatment. *Bapx1* expression is thought to be joint promoting in the mandibular arch [19]. Normally, *bapx1* function keeps the region between Meckel’s cartilage and palatoquadrate chondrocyte-free and enables the formation of the jaw joint. In perturbed specimens with elevated and expanded expression of *bapx1*, cartilage development was prevented within the muscular process of the palatoquadrate. The prevention of cartilage development led to the establishment of additional chondrocyte-free regions. Such a region separates numerous chondrocytes from their primordial cartilage. These chondrocytes can then condense and develop into a new cartilage.

Thus, our results show that Ly-294,002 treatment led to simultaneous upregulation of *bapx1* and development of an ectopic cartilage. *Bapx1* may be able to prevent cartilage formation in a restricted area and this prevention might subdivide existing cartilages. We suggest that *bapx1* promotes the formation of cartilage free regions within existing cartilages, which could lead to the formation of cartilage-free region within an existing cartilage and/or the formation of an ectopic cartilage. This function could be a foundation for the emergence of novel cartilages during anuran evolution, and it suggests that new cartilages can arise during development, and therefore during evolution, by subdivision of existing cartilages.

### The evolution of adrostral cartilages might be caused by changed *bapx1* expression

It has been suggested that *bapx1* was involved in the evolution of the gnathostome jaw joint. *Bapx1* was co-opted into the first arch in gnathostomes and replaced *barx1* in the ventral-intermediary region of the mandibular arch [1]. *Barx1* has been shown to repress joint formation and promote cartilage formation, whereas *bapx1* promotes joint formation and represses cartilage formation [19]. Thereby, *barx1* function in the agnathan mandibular arch ensures that no joint develops within this arch. The replacement of *barx1* by *bapx1* and thus the expression of *bapx1* in the ventral intermediary region of the mandibular arch might have caused the development of a joint within this arch in gnathostomes. This potential of *bapx1* to prevent cartilage formation and subdivide existing cartilages might be the explanation for the evolution of additional cartilages in larval anurans. The larval anuran jaw consists of Meckel’s cartilage and the infrarostral in the lower jaw. The upper jaw is formed by the paired cornua trabeculae and a plate-like suprarostral. Both rostralia are unique in anurans but no *bapx1* expression can be seen during development in the region of the rostralia or their respective precursors. Additionally, *bapx1* knockdown has no effect on the formation of the rostralia in *X. laevis* (unpublished).

**Fig. 8:**
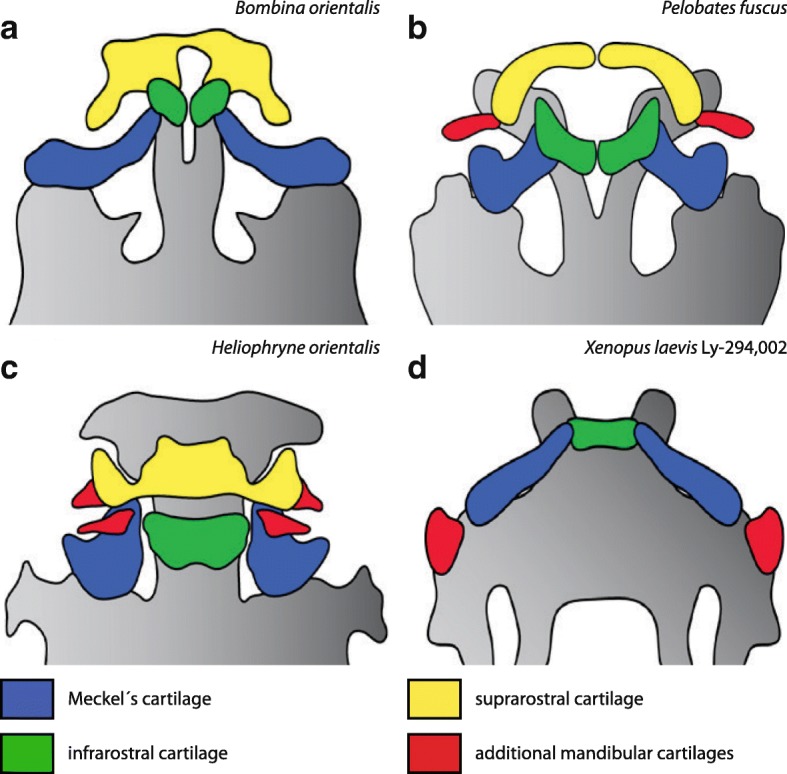
Overview of the diversity of adrostral cartilages among anurans. Ventral view of the jaw region of selected anuran larvae. **a** *Bombina orientalis* shows a typical anuran condition of the jaw region consisting of paired infrarostral cartilages, a plate-like suprarostral cartilage and horizontally oriented Meckel’s cartilages. **b** In *Pelobates fuscus* (drawn after Nikitin 1986) two so-called adrostral cartilages can be observed lateral to the paired suprarostral cartilages. **c** *Heliophryne orientalis* possesses two additional cartilages in the lower jaw. The submeckelian cartilages lie beneath Meckel’s cartilages and the adrostral cartilages are situated lateral to the suprarostral cartilage. In Ly-294,002-treated *Xenopus laevis* larvae (**d**) the ectopic cartilages arise lateral to the palatoquadrate and are free from any muscle insertion or origination similar to *P. fuscus* and *H. orientalis*

Several anurans, such as *Alytes obstetricans* [4], *Heliophryne purcelli* [38] and *Pelobates fuscus* [39], have been described in which one or more paired additional cartilages develop during the tadpole stage. Their shape and location within the jaw is different in the different species. They can appear as dorsoventral proceeding rods lateral to the suprarostral cartilages, as in *Pelobates fuscus* (Fig. 8b), or as cuneiform cartilages ventral to Meckel’s cartilage and lateral to the suprarostral cartilage, as in *Heliophryne orientalis* (Fig. 8c). In both cases they are located laterally within the lower jaw. The ectopic cartilages in Ly-294,002 treated *X. laevis* specimen are also located laterally, but more posteriorly (Fig. 8d). Our observations on *Heliophryne orientalis* tadpoles have shown that both additional cartilages, the sub-meckelian cartilage and the adrostral cartilage, lack muscle insertion or origination. The muscle-free sub-meckelian and adrostral cartilages are similar to the muscle-free condition of the ectopic cartilages in *A. mexicanum* and *X. laevis* treated with Ly-294,002. Both the absence of musculature and the lateral position of the additional cartilages are shared similarities between the naturally occurring adrostral cartilages and the ectopic cartilages which develop after Ly-294,002 treatment. These similarities and the ability of *bapx1*, whose upregulation is correlated to the development of the ectopic cartilages after Ly-294,002 treatment, to subdivide existing cartilages indicate that changes in *bapx1* expression (upregulation or a heterotopic shift) may have been the reason for the evolution of novel cartilages in the mandibular arch within anurans. Expression analysis of *bapx1* in the appropriate species can be used to test this hypothesis.

## Conclusion

In the present study we treated two amphibian species with Ly-294,002, and found that such treatment increased *bapx1* expression and caused mandibular arch-derived ectopic cartilages to develop lateral to the palatoquadrate in the larvae. The appearance of additional cartilages, which develop through separation from pre-existing cartilages, simultaneously to *bapx1* upregulation supports the notion that *bapx1* has a joint-promoting function. The putative function further substantiates a possible role for *bapx1* in the evolution of the gnathostome jaw joint. Additionally, *bapx1* function may explain the development of additional cartilages in the anuran jaw, which could be caused by overexpression of *bapx1* or by a heterotopic shift of its expression domain. The development of the ectopic cartilages implies that subdivision of pre-existing structures through changes in the expression of a developmental regulator is one possible contributing event in the evolution of morphological novelties.
